# Annual Banquet of the Atlanta Society of Medicine

**Published:** 1890-07

**Authors:** 


### ANNUAL BANQUET OF THE ATLANTA SOCIETY OF MEDICINE.

---

On the evening of the 18th instant, at Donehoo's restaurant, was held the fifth annual banquet of the Atlanta Society of Medicine.

The dinner was one of the most delightful in the history of the Society, and was participated in by almost every member of the organization. The entertainment reflected much credit on both caterer and Entertainment Committee, and especially so upon Dr. Cooper, the efficient chairman of that committee. The first, toast of the evening was "The Atlanta Society of Medicine," by Dr. William Perrin Nicolson. Dr. Nicolson, after reviewing, briefly, the good work already accomplished by the Society, urged the members to an increase of interest and more constant attendance. He also spoke of the necessity for a better place in which to hold the meetings, and pledged himself to procure a more desirable hall during the summer vacation.

Dr. Todd next responded to the toast the "Medical Profession" in his usual happy style. Then followed a number of excellent impromptu speeches by most of the gentlemen present.

The effects of these social meetings are quite apparent in Atlanta. The old bickerings and strifes which once disgraced the profession here have been succeeded by a feeling of mutual respect and common interests and aims.

There are a number of prominent physicians in the city who are not members of the society, and they should be made to feel that it is a duty and privilege which they cannot afford to neglect.

---

OUR PREMIUM LIST.—We wish to call especial attention to our premium list which appears in this issue of the JOURNAL.

Dr. Jas. B. Baird has been elected to the chair of General Medicine, in the Southern Medical College, left vacant by the resignation of Dr. W. D. Bizzell. We congratulate the institution on securing the services of a gentlemen so eminently fitted for the place.

---

COMPLIMENTARY TO THE JOURNAL.—The letter which we present below shows in what esteem the JOURNAL is held by one of the most prominent advertising agents in the North.

*Dr. M. B. Hutchins*, "*Atlanta Medical and Surgical Journal*,"
*Atlanta*, *Ga.*:

MY DEAR DOCTOR—As you possibly know I never let an opportunity go by to tell my advertising friends that the "ATLANTA MEDICAL AND SURGICAL JOURNAL" is the best journal in your section of the country, and to my knowledge I know this has influenced quite a number of contracts in your favor.

I expect in the fall to figure on placing the advertisements in various journals, of several advertisers, and it would be my pleasure to include your journal in any estimate I may make, because I confidently believe that you can give my advertisers very much better service than any other journal published in the South.

---

J. B. Lippincott Company announce in press an important work on "REGIONAL ANATOMV in its relation to MEDICINE AND SURGERY" by George McClellan, M. D., Lecturer on Descriptive and Regional Anatomy at the Pennsylvania School of Anatomy; Professor of Anatomy at the Pennsylvania Academy of the Fine Arts; member of the Association of American Anatomists, Academy of Natural Science, Academy of Surgery, College of Physicians, etc., of Pennsylvania. With about 100 full-page fac-simile illustrations reproduced from photographs taken by the author of his own dissections; expressly designed and prepared for this work, and colored by him after nature. To be complete in two volumes of 250 pages each. Large quarto.

The object of the work is to convey a practical knowledge of Regional Anatomy of the entire body. The text to embrace, besides a clear description of the part in systematic order, the most recent and reliable information regarding anatomy, in its medical and surgical relations. The illustrations are intended to verify the text and to bring before the reader the parts under consideration in as realistic a manner as possible. Vol. I will be ready for publication about December 1st, and the second volume is expected to appear shortly thereafter. The work will be sold by subscription only; and salesmen will begin an active canvass the coming October.

